# Repurposing current therapeutic regimens against SARS-CoV-2 (Review)

**DOI:** 10.3892/etm.2020.8905

**Published:** 2020-06-18

**Authors:** Sofia K. Konstantinidou, Ioannis P. Papanastasiou

**Affiliations:** 1 Oncology Unit, The Third Department of Medicine, Medical School, National and Kapodistrian University of Athens, 11527 Athens, Greece; 2 Division of Pharmaceutical Chemistry, Department of Pharmacy, School of Health Sciences, National and Kapodistrian University of Athens, 15784 Athens, Greece

**Keywords:** SARS-CoV-2, COVID-19, pandemic, mechanism of action, clinical trials, vaccines

## Abstract

The recent coronavirus outbreak has spread worldwide, with the exception of Antarctica, causing serious social and economic disruption. All disciplines of the science community are driven to confront the impact of the COVID-19 pandemic, as currently, there is neither prophylactic nor therapeutic treatments available. Due to the urgency of the situation, various research strategies are ongoing, in order to evaluate the therapeutic efficacy of repurposed and experimental drugs. The present review presents the most promising repurposed regimens, which may be used for the treatment of COVID-19. The drugs/bioactive substances presented herein belong to diverse therapeutic classes, including antimalarial, cardioprotective, angiotensin-converting enzyme 2 inhibitors, antiviral, anti-inflammatory and antiparasitic drugs. Therapeutic perspectives of vaccination and passive immunization are also reviewed.

## 1. Introduction

**Figure 1:**
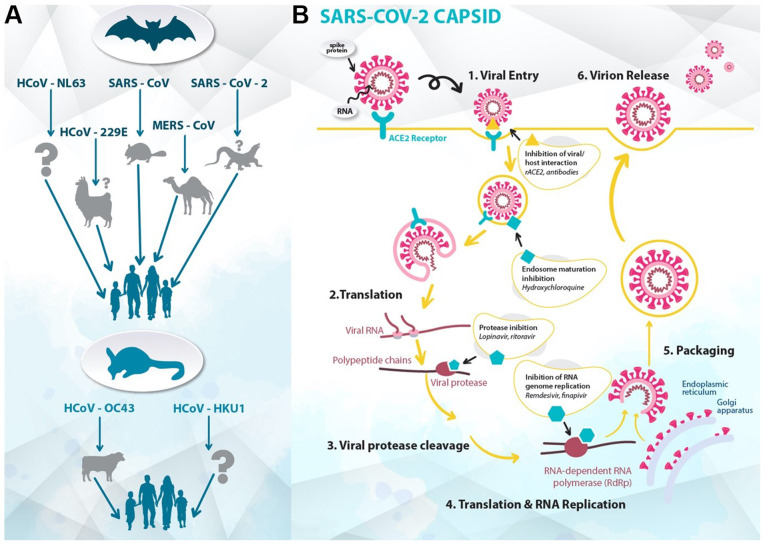
(A) Animal hosts of coronaviruses. Arrows depict the transmission of different coronavirus strains from their natural hosts (bats or rodents), through the intermediate hosts (camelids, civets, dromedary camels, pangolins or bovines), to humans. (B) Stages and targets of the life cycle of SARS-CoV-2 in host cells. SARS-CoV-2, severe acute respiratory syndrome coronavirus 2; HCoV, human coronavirus; MERS, Middle East respiratory syndrome; ACE2, angiotensin-converting enzyme 2.

The outbreak of the 2019 novel coronavirus in Wuhan, China, spread worldwide, becoming a pandemic in the beginning of 2020(1). The World Health Organization (WHO) announced the name of the disease to be coronavirus disease-19 (COVID-19), and the Coronaviridae Study Group of the International Committee on Taxonomy of Viruses classified its etiological agent as severe acute respiratory syndrome coronavirus 2 (SARS-CoV-2) (Fig. 1) (2). This respiratory pathogen belongs to the viral family Coronaviridae, which was first identified in the mid-1960s (3,4). Mutation and adaptation have driven the co-evolution of coronaviruses and their hosts, including humans, for thousands of years (5). Recently, in 2002-2003, the outbreak of severe acute respiratory syndrome coronavirus (SARS-CoV) increased the awareness of the fatal risk and medical importance of virulent coronavirus strains (6). In 2012, a novel zoonotic coronavirus related to SARS was identified in the Middle East, known as Middle East respiratory syndrome coronavirus (MERS-CoV), which had a mortality rate of 36% (7). Phylogenetic analysis revealed that SARS-CoV-2 is quite distant from SARS-CoV (~79%) and MERS-CoV (~50%) (8). Compared with the latter two strains, SARS-CoV-2 has a lower case-fatality rate; however, it spreads more efficiently (9). In response to the emergence of the pandemic, a third of the global population was set on lockdown, and the authorities implemented measures to slow the spread of the infection, from mandatory geographic quarantines to non-mandatory recommendations. At present, as there is neither prophylactic nor therapeutic treatment for COVID-19, the world has accelerated research on treatments and vaccines for this threat (10). Due to the urgency of the situation, a number of already approved and marketed drugs are being tested for repurposing, conforming to a recent strategic innovation in medicinal chemistry and drug discovery (11).

## 2. Currently available drugs against SARS-CoV-2

### 

#### Antimalarial regimen: Chloroquine and hydroxychloroquine (Fig 2)

Chloroquine and its derivative hydroxychloroquine have previously been used as clinical treatments in various diseases (12-14). Chloroquine hydrochloride and phosphate, and its derivative, hydroxychloroquine sulfate, have been commercialized for the prophylaxis and treatment of malaria, chronic Q-fever and autoimmune diseases (15). Both chloroquine and hydroxychloroquine are available as racemates, which may exhibit stereoselective metabolism and efficacy (16). Chloroquine is a 4-aminoquinoline analogue that acts effectively as a schizonticidal drug against the erythrocytic forms of all types of plasmodia (17). It has also been reported to have modest results against chronic hepatitis C (12). Numerous mechanisms of action have been suggested to clarify the activity and the side effects of chloroquine and hydroxychloroquine, most of them implicating lysosomal activity on a molecular level (18), autophagy and signaling pathways (19), and/or immunomodulatory potency, by reducing anti-inflammatory cytokines (20). This observation might lead to a new perspective and the therapeutic consideration of lysosomotropic agents and/or sigma receptor(s)-related drugs for the treatment of COVID-19 (21,22).

#### In vitro

studies have revealed that chloroquine is highly effective against SARS-CoV-2. In an *in vitro* study by Keyaerts *et al* (23), it was shown that chloroquine exhibited antiviral activity with an IC50 of 8.8 M. Wang *et al* (24) also reported that the 90% maximal effective concentration value of chloroquine against SARS-CoV-2 in Vero E6 cells was 6.90 µM, thus being potentially clinically applicable. In addition, hydroxychloroquine was tested *in vitro* by Liu *et al* (25), where it was found to efficiently inhibit SARS-CoV-2 by blocking the entry step of the virus, as well as its post-entry stages. Until new medications are approved, chloroquine phosphate or its derivative, hydroxychloroquine, in combination with azithromycin and/or antimicrobial therapy, are being adjuvantly used for patients with COVID-19 in various countries, such as France, Greece, China and USA (26,27). The first results on humans were obtained from Chinese hospitals, which revealed the superiority of chloroquine compared with the control group, as it reduced the exacerbation of pneumonia, duration of symptoms and delayed viral clearance (27,28). Therefore, China recommended chloroquine for the treatment and prevention of COVID-19(14). Patients with COVID-19 pneumonia may benefit from the anti-viral and anti-inflammatory action of chloroquine (29).

As well as treating malaria, chloroquine and hydroxychloroquine have been used in the past against various rheumatic diseases, including systemic lupus erythematosus and rheumatoid arthritis. Both drugs exhibit their antiviral action in a short time after their administration and cause immune modification, as they reduce the production of cytokines (14). With regards to the reduced production of proinflammatory cytokines, these drugs mainly affect IL-1 and IL-6, and inhibit the activation of Toll-like receptors (TLRs) (30).

An investigation led by Professor Didier Raoult recently released the results of a new hydroxychloroquine treatment study on 1,061 patients in Marseille, France (26). In this cohort study, the patients were administered a hydroxychloroquine-azithromycin (HCQ-AZ) combination, for ≥3 days, and then were followed-up for ≥9 days. HCQ-AZ led to a good clinical outcome within 10 days of treatment (91.7%) and mortality was lower in patients who had received the HCQ-AZ combination for >3 days. This study revealed that HCQ-AZ in the early phases of mild COVID-19 could prevent exacerbation of the infection; however, it still remains a question if it has any effects on severe cases (26).

The optimal dosages of chloroquine and hydroxychloroquine, as well as the required duration of administration for the treatment of COVID-19, have not been determined, and several clinical studies have used different dosing regimens (31-33). The ongoing PATCH Trial (34) (randomized study with 400 participants) aims to compare different dosage forms of hydroxychloroquine in terms of their effectiveness against COVID-19. However, for patients with renal or hepatic impairment, there are no specific dosage recommendations, except for the advice ‘use with caution’. Treatment with chloroquine can lead to severe adverse effects and overdosing can lead to pulmonary edema and circulatory collapse, which are even more severe in the elderly, thus dose reduction is recommended (35).

Particular care is required in the use of chloroquine phosphate, hydroxychloroquine and azithromycin, due to their cardiotoxicity and QT prolongation (35). A multinational cohort study conducted by Lane *et al* (36) indicated that when azithromycin was added to hydroxychloroquine, patients exhibited increased risk of 30-day cardiovascular mortality, chest pain and heart failure. Therefore, due to the potentially synergistic effects of HCQ-AZ on QT length, caution should be taken. Patients with chronic diseases, including kidney failure, liver disease, epilepsy and myasthenia gravis, or patients on medications that include active substances incompatible with these drugs, will require strict monitoring because they are likely to cause serious arrhythmias. Other risk factors that can cause arrhythmias (e.g. torsades de pointes) include hypomagnesaemia, hypokalemia, bradycardia, heart failure, advanced age and QT >450-500 msec. In addition, chronic treatment or high doses of chloroquine and hydroxychloroquine may lead to damage to the retina. It is also important that checks for lack of the G6PD enzyme are conducted, as lack of this enzyme is associated with the toxicity of these drugs, mainly leading to retinopathy (37).

Recently, a multinational worldwide study conducted on patients hospitalized with COVID-19 revealed that the use of hydroxychloroquine or chloroquine (in combination with or without a macrolide) was associated with no evidence of benefit, but instead was associated with an increased risk of ventricular arrhythmias and a greater hazard for in-hospital mortality (38). However, the article was retracted as several concerns were raised with regards to the validity of the patients' data. Currently, there is no effective and safe chloroquine dosage treatments for COVID-19. Karalis *et al* (39) used simulation techniques for optimization of dosage regimens and suggested specific recommendations to healthcare specialists.

#### Targeting cardioprotective derivatives: Colchicine

COVID-19 has various cardiovascular implications, in particular : i) Patients with COVID-19 and pre-existing cardiovascular disease exhibit a high risk of severe disease and death; ii) COVID-19 has multiple direct and indirect cardiovascular complications; and iii) currently used therapies against COVID-19 may have cardiovascular side effects. For all these reasons, colchicine has been proposed to be added to the therapeutic regimen against SARS-CoV-2(40). This drug has been used for several years against diseases such as gout, Mediterranean fever and pericarditis, and in addition to its other properties, it has exhibited anti-inflammatory action (41). COLCORONA (42) is a multi-center, randomized, double-blind trial with 6,000 participants that is currently in progress, which aims to investigate whether short-term treatment with colchicine may reduce mortality and lung complications in patients with COVID-19. Moreover, GRECCO-19(41) is a smaller randomized clinical trial, with an estimated 180 participants, which was launched in Greece with the aim of identifying the role of colchicine in patients with COVID-19 and determining whether it has an effect on the clinical course of COVID-19 by reducing myocardial necrosis and pneumonia (41).

#### Targeting angiotensin-converting enzyme 2 (ACE2) (Fig 3)

The entry of SARS-CoV-2 into cells is associated with the binding of the viral spike S protein with ACE2(43). A defined receptor-binding domain in the aforementioned spike protein specifically recognizes its host receptor on ACE2. Both coronavirus strains, SARS-CoV and SARS-CoV-2, share a high sequence identity in spike S protein (~76%) (44). This similarity of receptor recognition may be a major factor associated with host range and cross-species mutations, and with the emergence of coronavirus infections (45). ACE2 is a zinc metalloproteinase and is part of the renin-angiotensin system (RAS), which maintains cardiovascular homeostasis and regulates blood pressure through electrolyte balance (46). ACE2 hydrolyzes the carboxy-terminal leucine from angiotensin I (decapeptide) to produce the nonapeptide angiotensin-(1-9). ACE2 also converts angiotensin II (octapeptide) to angiotensin-(1-7), which induces blood vessel relaxation, and anti-proliferative and anti-oxidative stress activities. Regarding coronavirus infections, ACE2 mediates S protein-driven host cell entry (47,48). Moreover, the viral spike S protein has been suggested to downregulate ACE2 expression in host cells, thus leading to severe lung injury and acute lung failure (49). The formation of the S protein-ACE2 complex has also been reported to lead to a partial decrease or total loss of the enzymatic ACE2 function in these cells, thus increasing the tissue concentration of proinflammatory angiotensin II. This is affected by decreasing its degradation and reducing the concentration of angiotensin-(1-7), which is its physiological antagonist. High levels of angiotensin II in the lung interstitium can promote apoptosis, thus initiating an inflammatory process and resulting in the release of proinflammatory cytokines, which may eventually lead to acute respiratory distress syndrome (ARDS) (50).

The therapeutic approaches (51) against ACE2-mediated COVID-19 may involve: i) Vaccine production based on the spike S protein (52), ii) inhibition of virus and host cell fusion via transmembrane protease serine 2 (TMPRSS2) (53,54) and iii) blocking ACE2 receptor (55,56).

Another valuable regulating factor of the RAS with regards to COVID-19 are angiotensin II receptor blockers (ARBs), previously described as angiotensin II type 1 (AT1) receptor antagonists. Initially, it was hypothesized that patients treated with ARBs or ACE inhibitors would be at a higher risk of new coronavirus infection (57,58). This is due to the fact that expression of ACE2, which is the enzyme that SARS-CoV-2 binds to, is increased in patients treated with ARBs or ACE inhibitors (57). ARBs directly protect the endothelial barrier integrity of the lungs and indirectly upregulate ACE2, reducing inflammation, organ fibrosis and endothelial injury (56). Among these blockers, losartan has been suggested to be a drug candidate as it can strongly bind to AT1(59). Notably, Zhang *et al* (60) revealed that hospitalized patients with COVID-19 and hypertension, when treated with ACE inhibitors or ARBs, exhibited a lower risk of all-cause mortality compared with non-users. According to Rothlin *et al* (50), out of all of the ARBs available, telmisartan may have best pharmacological properties to be evaluated for COVID-19.

It has also been reported that statins upregulate ACE2 via epigenetic modifications and interfere with its signaling. Castiglione *et al* (61) hypothesized that adjuvant treatment or continuation of already existing statin therapy could improve the clinical course of patients with COVID-19. This finding may be due to their immunomodulatory action or by the prevention of cardiovascular damage (62). Alongside their lipid-lowering activity, statins may reduce inflammation and oxidative stress, thus contributing to their beneficial action on cardiovascular diseases. In addition, statins intervene in the immune response at different levels, including immune cell adhesion and migration, and cytokine production (61). Statins also restore the vascular redox balance by reducing reactive oxygen species, and ameliorating nitric oxide bioavailability, endothelial function and integrity (61). Statins, alongside ARBs, have been reported to be effective in targeting the host response and preventing endothelial barrier damage in patients infected with the Ebola virus during the recent Ebola outbreak in West Africa (63). These findings propose a research direction against the SARS-CoV-2 virus (64).

#### Targeting antivirals Remdesivir (Fig. 4)

Experimental nucleosides and approved nucleoside analogues have been used against SARS-CoV-2(65). Remdesivir (GS-5734) is a monophosphoramidate analogue of adenosine with potency against Ebola virus (66). The 1'-cyano-substituted adenine ribose analogues have exhibited significant activity against RNA viruses (67). In addition, *C*-nucleosides are more stable than the natural and synthetic *N*-nucleoside congeners, which are vulnerable to enzymatic and acid-catalyzed hydrolysis of the nucleosidic bond (68). GS-5734 has to be anabolized intracellularly to the active triphosphate metabolite, which acts as a chain terminator of viral RNA-dependent RNA-polymerases. The parent nucleoside is modified by a monophosphate prodrug moiety of the 2-ethylbutyl *L*-alaninate and enhances the intracellular triphosphate metabolite concentrations, bypassing the rate-limiting first phosphorylation kinetics (69).

Remdesivir has a broad-spectrum of antiviral activity against several emerging viral pathogens, including Ebola, Marburg, MERS and SARS. *In vitro* testing conducted by Gilead Sciences, Inc. demonstrated that remdesivir may be active against the SARS-CoV-2 virus. Currently, the safety and efficacy of remdesivir for the treatment of COVID-19 are being evaluated in numerous ongoing phase III clinical trials (70). However, although several antiviral drugs are being tested against SARS-CoV-2 *in vitro*, no drugs are currently available with proven effectiveness in patients with severe COVID-19 infection (71).

A cohort study by Grein *et al* (72) on hospitalized patients with severe COVID-19 who were treated with compassionate use of remdesivir revealed that improvement was observed in 68% of patients in the respiratory support category (ambient air, non-invasive oxygen support, invasive mechanical ventilation and extracorporeal membrane oxygenation). In another randomized, double-blind, multicenter trial, the Adaptive COVID-19 Treatment Trial (ACTT) (73), the antiviral drug remdesivir was compared with a placebo in hospitalized patients with severe COVID-19 symptoms. At present, ACTT results from patients treated with remdesivir compared with a placebo had a 31% faster time to recovery (median time to recovery: 11 vs. 15 days). Other clinical trials are under way to assess the safety and efficacy of remdesivir (74,75).

Gilead Sciences, Inc. has initiated two randomized, open-label, multi-center phase III clinical trials for remdesivir, the SIMPLE trials (74,75), in countries with a high prevalence of COVID-19. The first aims to evaluate the safety and efficacy of 5- and 10-day dosing regimens of intravenous remdesivir in hospitalized patients with severe manifestations of COVID-19, and the second in patients with moderate symptoms of COVID-19, compared with standard of care. According to Gilead Sciences, Inc., in an exploratory analysis, 10-day treatment with remdesivir achieved similar improvement in the clinical status of patients compared with a 5-day treatment course (70).

#### Favipiravir (Fig. 5)

Favipiravir (T-705) is a guanine analogue against influenza, which was approved in Japan in 2014. T-705 inhibits the RNA-dependent RNA polymerase of RNA viruses, such as influenza, Ebola, yellow fever, chikungunya, norovirus and enterovirus (76), and was recently quite successfully tested against SARS-CoV-2(65). Favipiravir undergoes intracellular phosphoribosylation to form the active ribofuranosyl triphosphate metabolite (77,78).

Favipiravir has shown encouraging results in Chinese clinical trials; in particular, it accelerated the recovery of hospitalized patients and improved lung function. It has a broad spectrum of activity against RNA viruses, but not against DNA viruses (76). Cai *et al* (79) compared favipiravir with lopinavir/ritonavir for the treatment of COVID-19 in a non-randomized clinical trial, and revealed that favipiravir had a greater effect on viral clearance and greater improvements on chest computed tomography scans. In addition, Chen *et al* (80) designed a multi-center randomized superiority clinical trial of favipiravir versus umifenovir for the treatment of patients with COVID-19 in Wuhan, China (77). It was revealed that favipiravir significantly improved pyrexia and cough, but did not significantly improve the clinical recovery rate at day 7. Currently, this drug is not available in Europe; however, there are ongoing randomized clinical trials (81-84), which aim to evaluate the role of favipiravir in patients with COVID-19; further results are expected after May, 2020.

#### Ribavirin

Ribavirin is a purine nucleoside analogue that is used to treat numerous viruses, such as respiratory syncytial virus and hepatitis C (85). Ribavirin prevents the replication of viruses by inhibiting the cellular enzyme inosine monophosphate dehydrogenase (86). Additionally, the 5'-triphosphate metabolite of ribavirin inhibits viral polymerase activity.

Although ribavirin has been reported to have little inhibitory effect on coronavirus replication, it decreased the production of proinflammatory cytokines, such as IL-1 and TNF-α, in the macrophages of mice (87). Ribavirin exhibits immunomodulatory potency as well as antiviral activity, and has already been used against SARS-CoV and MERS-CoV (85). Ribavirin is being tested in clinical trials against COVID-19 and has shown promising results when used as a triple combination with interferon (IFN)β-1b and lopinavir-ritonavir (88).

#### HIV protease inhibitors: Kaletra (lopinavir/ritonavir)

Lopinavir and ritonavir are HIV protease inhibitors (89), which have been specifically designed to fit a certain HIV protease dimer pocket that is not present in coronavirus proteases. The combination of lopinavir/ritonavir was initially thought to be a promising combination against COVID-19, based on its mechanism of action, but ultimately did not prove useful in combating COVID-19. Initially, significant antiviral activity of lopinavir/ritonavir against SARS-CoV was reported in cell cultures; however, there were conflicting results for MERS-CoV (90). A randomized, controlled, open-label trial by Cao *et al* (91) revealed that the combination of lopinavir/ritonavir in hospitalized adult patients with severe COVID-19 had no benefit compared with the control group. No significant effect was detected on primary outcome and time to clinical improvement (HR, 1.31); in addition, mortality at 28 days was similar in the lopinavir/ritonavir group compared with the standard-care group (19.2 vs. 25.0%) (91).

Another ongoing multicenter randomized clinical trial (currently in phase II; estimated completion: May, 2020) aims to compare the lopinavir/ritonavir combination with hydroxychloroquine in patients with mild COVID-19, and will investigate whether these treatments reduce the viral load from the respiratory specimens of these patients (32). More randomized clinical trials (92,93) are still ongoing, and the results will soon become available. Recently, the results of a multicenter randomized phase II trial were published, which compared a 14-day triple combination of lopinavir/ritonavir, ribavirin and IFNβ-1b with lopinavir-ritonavir alone, for the treatment of hospitalized patients with mild to moderate COVID-19(94). The results revealed that early triple antiviral combination improved the symptoms of patients compared with lopinavir/ritonavir alone; it also reduced the duration of hospitalization (91). Another possible treatment includes homoharringtonine, which has been reported to be active against herpes viruses, coronaviruses and rhabdoviruses (95). An *in vitro* study reported that homoharringtonine inhibited SARS-CoV-2 (half maximal effective concentration, 2.10 µM); therefore, it could serve as a potential agent to be used in clinical trials for COVID-19(96). The combinational therapies of the aforementioned drugs may decrease the effective concentration of the antivirals below therapeutic plasma concentrations and provide better clinical benefits (96).

#### Targeting proinflammatory hypercytokinemia: Tocilizumab and leronlimab

Increased death rates have been noted in patients with certain risk factors, due to an overwhelming reaction of the immune system to the virus, causing hypercytokinemia (termed cytokine storm), i.e. cytokine-release syndrome (CRS) and macrophage activation syndrome (MAS), which can result in ARDS (97). CRS has been seen in response to coronaviruses, including SARS and MERS, with a high expression of IL-6. Several research reports have demonstrated that some proinflammatory cytokines are increased in the plasma of patients with COVID-19 (96,97). Therefore, it was suggested that cytokine-targeted biological therapies may improve outcomes in CRS or MAS (97).

Therapies that are being investigated for the treatment of patients with COVID-19 with severe respiratory distress, symptom exacerbation and hyperinflammatory syndrome include monoclonal antibodies, such as tocilizumab (IL-6 antagonist), leronlimab (C-C chemokine receptor type 5 inhibitor) and Janus kinase inhibitors (98,99). The CORIMUNO-19 clinical trial platform was designed and developed quickly to evaluate the efficacy and safety of various immune system regulators, as well as other treatments in adult patients with severe COVID-19 infection (100). The primary outcome was the need for ventilation or death at day 14; the results revealed that a significantly lower proportion of patients in the tocilizumab arm reached the primary outcome (101).

IL-6 is a cytokine that is thought to increase even further the inflammation in COVID-19, thus leading to the production of more cytokines, macrophages and cytotoxic lymphocytes that increase lung inflammation. Increased IL-6 levels have been reported to be related to ARDS (102). Tocilizumab, an anti-IL-6 receptor (IL-6R) biological therapy, has been approved for the treatment of CRS and is also used to treat patients with MAS. A small clinical trial from China exhibited good efficacy in patients with COVID-19 treated with tocilizumab (103). Phase II trials (104,105) have shown that tocilizumab, by blocking IL-6R, may reduce inflammatory markers in patients with COVID-19 and accelerate clinical improvement.

COVACTA (106) is a phase III randomized clinical trial, which aims to evaluate intravenous tocilizumab combined with standard of care, compared with a placebo plus standard of care in patients with severe COVID-19 pneumonia. Early data from a clinical trial in France has shown encouraging results of tocilizumab in critically ill patients with COVID-19(107). The National Health Commission of China has also suggested the use of tocilizumab in patients with COVID-19 with extensive bilateral lung lesions opacity or in critically ill patients with increased IL-6 levels (102).

#### IFNλs

IFNλs are crucial for maintaining a balanced antiviral response in the respiratory tract, by inducing viral resistance to cells and helping them deal with the virus load (108). Recombinant or pegylated forms of IFNλ can prevent a cytokine storm and reduce viral load, thus preventing lung tissue damage (109). IFNλ may be a promising therapy for the treatment of patients with COVID-19, which should be tested in clinical trials, as it may prevent severe pneumonia symptoms and ARDS, and thus death. Lung inflammation and tissue damage are some of the severe signs of COVID-19 infection, which arise due to cytokine storm (110). However, it still needs to be evaluated whether, by stimulating IFNλs or if severe inflammation is present, they can be upregulated and thus increase the possibility of adverse effects on humans (111).

#### Antiparasitics

Ivermectin, an antiparasitic agent by Merck Sharp & Dohme, is the generic name given to a mixture of two chemically modified avermectins containing ≥80% 22,23-dihydroavermectin B_1a_ and <20% of the corresponding B_1b_ homologue. Avermectins are macrolide antibiotics derived from the fermentation products of the actinomycete *Streptomyces avermitilis* (112). Ivermectin demonstrated *in vitro* inhibitory action against the replication of SARS-CoV-2(113). Ivermectin has an established safety profile for human use and has been approved for the treatment of several parasitic infections (114,115). Therefore, it is worthy of further consideration as an antiviral agent against SARS-CoV-2. Furthermore, nitazoxanide, a US Food and Drug Administration-approved drug with a broad spectrum of antiparasitic and antiviral activity, has been suggested to be included in a new protocol for the early management of COVID-19, in combination with azithromycin (116). Nitazoxanide has already been repurposed for the treatment of influenza-like infections and may exhibit a synergistic effect with hydroxychloroquine. In addition, because nitazoxanide upregulates the innate immune response in order to prevent viral replication, it could reduce overall viral load of SARS-CoV2(117).

## 3. Miscellaneous

### 

#### Nicotine

Nicotine is a cholinergic agonist and an inhibitor of proinflammatory cytokines (118). Notably, nicotine inhibits TNF, IL-1 and IL-6, which are increased in patients with COVID-19 and are involved in the cytokine storm, leading to rapid deterioration (118). Thus, nicotine, by acting on the cholinergic anti-inflammatory system, has anti-inflammatory properties that may have a protective role in patients with COVID-19, by preventing the cytokine storm. Therefore, nicotine administration could be considered as an add-on therapy, alongside other medications, for the treatment of COVID-19. However, clinical studies are required in order to confirm this hypothesis.

#### Vitamin D

Vitamin D deficiency is a major problem worldwide and low serum levels of vitamin D may increase susceptibility to respiratory tract infections. Martineau *et al* (119) performed a systematic review and reported that vitamin D may protect against acute respiratory tract infections; people with a severe vitamin D deficiency experienced the most benefit from its administration. Vitamin D has been reported to prevent the release of increased inflammatory cytokines by changing the response of macrophages (120). Moreover, it has been demonstrated that infection with COVID-19 may lead to the release of proinflammatory cytokines, thus vitamin D may have a protective role against this (121).

#### Spironolactone

Recently, there have been some concerns about the use of spironolactone for the treatment of acne in patients with COVID-19 a small study from 2005 reported that macrophages taken from 10 patients with heart failure, who had been on spironolactone daily for a month, had increased ACE2 activity (122). However, spironolactone may have a protective action, as SARS-CoV-2 requires androgens to infect cells (123). In general, the entry of coronaviruses into host cells relies on binding of their viral spike S proteins to cellular receptors, and on S protein priming by host cell proteases. SARS-CoV-2 uses the receptor ACE2 for entry and the serine protease TMPRSS2 for S protein priming (53). Therefore, spironolactone, being an androgen blocker, may have a protective role against the virus, as well as having some lung and heart benefits (122). The lack of androgens could be a possible explanation for the rare cases of mortality among children with COVID-19, and the high number of male COVID-19-related fatalities in comparison to female fatalities (124). An ongoing randomized clinical trial aims to evaluate the role of spironolactone in COVID-19-induced ARDS (125).

## 4. Immunization

### 

#### Vaccines

As of May, 2020, there is no approved vaccine against COVID-19, but several are being tested in clinical trials (52). As well as research into vaccines against SARS-CoV-2, there have been some reports that Bacillus Calmette-Guérin (BCG), a vaccine against tuberculosis, may have a protective role against viral infections. Specifically, this vaccine affects the release of proinflammatory cytokines, which have a significant role in fighting viral infections (126). An epidemiological analysis revealed that countries with a BCG vaccination policy, such as Japan, had lower mortality rates compared with Italy, which does not have a BCG vaccination policy (127). BCG vaccination has also been shown to offer protection against viral infections and sepsis, by offering bystander immunity (128); therefore, it might have a positive impact against COVID-19. The BRACE and BADAS randomized trials, which are still recruiting patients, aim to determine whether the BCG vaccine reduces the incidence and severity of COVID-19 (129,130).

It has also been suggested that the Measles Mumps Rubella vaccine may provide a protective role against COVID-19, as it increases the ability of the immune system to fight infection (131). Moreover, there are some structural similarities between measles and COVID-19, which may cause cross-reactivity and thus protection against infection (131). However, for both vaccines, randomized clinical trials are essential in order to evaluate their role against SARS-CoV-2. There are currently several potent vaccines against COVID-19, which are being tested, some of which have already entered phase I.

#### Passive immunization

Current data have suggested that collected IgM and IgG antibodies from patients who have recovered from COVID-19 infection might be a therapeutic option for SARS-CoV-2, as they increase neutralizing antibodies (132). Until vaccines are discovered, passive immunization could be an alternative treatment strategy, particularly for high-risk groups, such as the elderly or patients with cancer (132). Keith *et al* (133) suggested the use of therapeutic plasma exchange (TPE) as a possible treatment for COVID-19 early in the clinical course, before patients proceed to septic shock or multiple organ failure. Plasma obtained from convalescent donors could be a possible therapeutic option against viral infections, an approach that has also been tried in the past (134). However, Honore *et al* (135) expressed serious doubts over the use of TPE, as it poses a risk in altering the host immune response. Notably, TPE may aggravate immunoparalysis, as it can remove protective antibodies from the patients and dilute the adaptive response to infection (135). Thus, the safety, efficacy and associated risks of TPE should be tested in randomized clinical trials.

## 5. Conclusions

In conclusion, the treatment regimens used to treat COVID-19, based on recent protocols, consist of known drugs that act against other diseases. The urgent need to treat this deadly pandemic requires the use of known drugs, at least until novel drugs and vaccines with specific/selective action against the virus are discovered. Four randomized trials have already been launched internationally in order to identify effective drugs, the first one for remdesivir (ACTT), the second and third for lopinavir/ritonavir [CATCO (136) and REMAP-CAP (137)] and the fourth for remdesivir and lopinavir/ritonavir, with or without IFNβ-1α and hydroxychloroquine [DisCoVeRy (138)]. The European Medicines Agency is in contact with those responsible for the development of twelve possible vaccines against COVID-19, and two vaccines have already been introduced in phase I clinical trials. Vaccine development schedules are difficult to predict, but it is estimated that it will take at least one year for a vaccine to be approved and be available in sufficient quantities in order to be widely used.

## Figures and Tables

**Figure 2:**
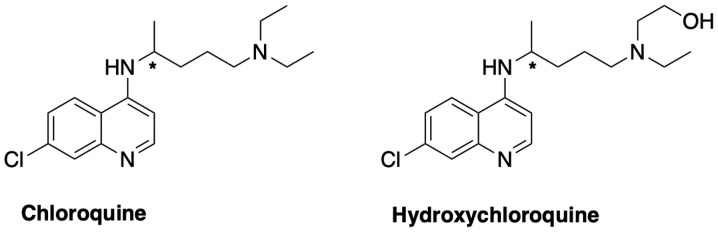
Structures of chloroquine and hydroxychloroquine.

**Figure 3:**
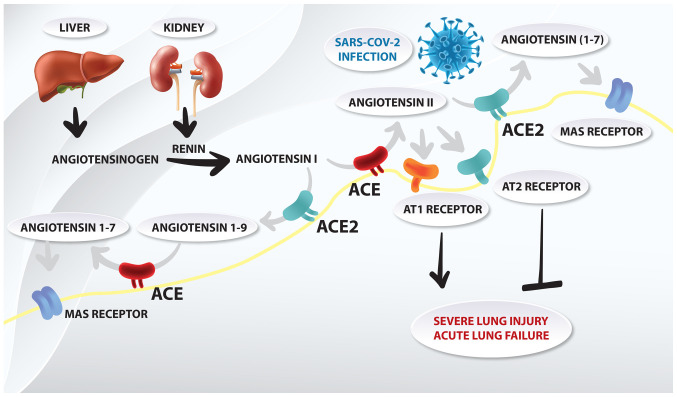
Schematic representation of the role of the renin-angiotensin system in acute lung failure of coronavirus disease 19. SARS-CoV-2, severe acute respiratory syndrome coronavirus 2; ACE2, angiotensin-converting enzyme; AT, angiotensin II receptor.

**Figure 4:**
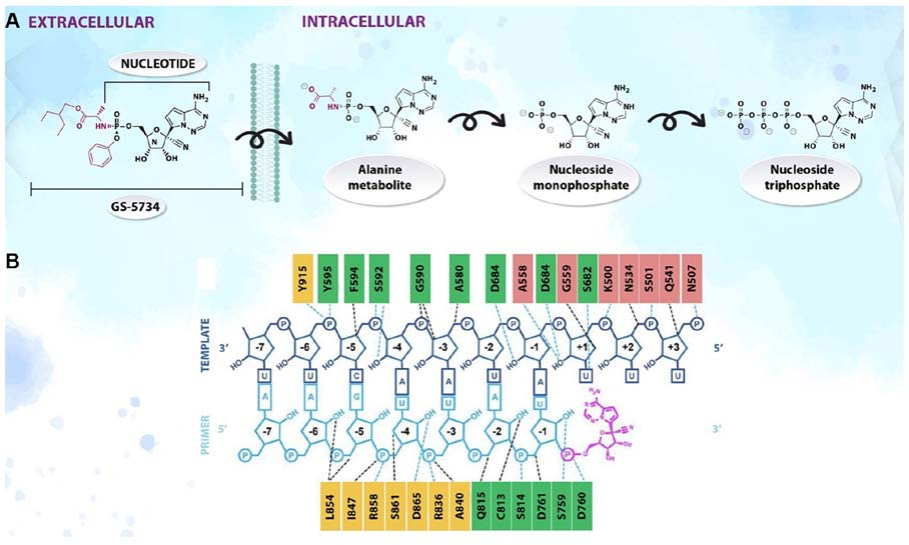
(A) Metabolism and (B) mechanism of action of remdesivir.

**Figure 5:**
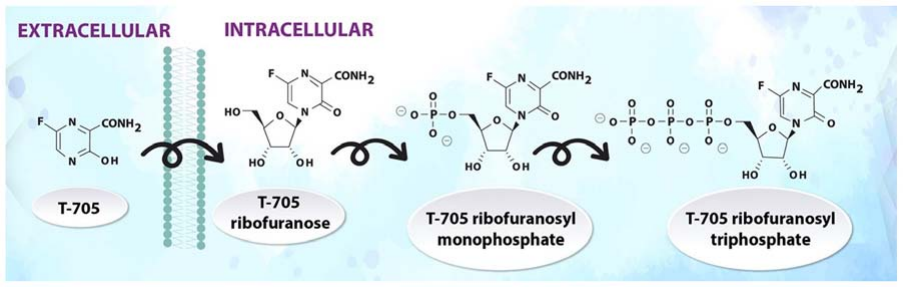
Metabolism of favipiravir.

## Data Availability

Not applicable.

## Authors' information

Miss Sofia Konstantinidou is an Onassis Foundation Scholar.
